# Asleep for Nine Years

**Published:** 1892-10

**Authors:** 


### ASLEEP FOR NINE YEARS AND STILL SLEEPING.

A remarkable case of suspended animation, of scientific interest, is referred to in an issue of the *English Mechanic*. The sleeper is a young woman named Marguerite Boyenval, of the village of Thenelles, in France, who fell into a cataleptic condition on May 29, 1883, since which day she has never shown the slightest sign of returning consciousness. When the actual nature of this profound sleep was realized by her medical attendants from the non-success which attended all efforts to awaken the young woman, attention was given to sustaining life. As the jaws were rigidly fixed, it was found to be impossible to introduce food into the stomach, and injections of nourishment were resorted to.

During these nine years she has been free from all cares of life, without thought or motion, consequently there has been no apparent waste of muscular or nervous tissue. The hand of time seems to have spared this unconscious sleeper, no change has taken place in her countenance, she appears no older to-day than when she fell asleep nine years ago at the age of twenty-five. During this time the growth of hair and nails has completely ceased ; the joints have become quite stiff from disuse, to such an extent that the arms, if raised, will remain in that position for an indefinite period.

The eyes are turned upward, so that the pupils are entirely out of sight on opening the eyelids. The lips, when moved apart, seem to lack the elasticity necessary to return to their original position.

